# Proliferative Glomerulonephritis With Monotypic Immunoglobulin Deposits: An Unusual Presentation in the Setting of Multiple Inciting Events Including COVID-19 Vaccination

**DOI:** 10.7759/cureus.25949

**Published:** 2022-06-15

**Authors:** Jagan Mohan Rao Vanjarapu, Jose Iglesias, Rumana Ahmed, Pratiksha Singh, Gabrielle Gerbino, Michael Barry Stokes

**Affiliations:** 1 Internal Medicine, Community Medical Center, Toms River, USA; 2 Nephrology, Community Medical Center, Toms River, USA; 3 Internal Medicine/Nephrology, Hackensack Meridian School of Medicine at Seton Hall University, Nutley, USA; 4 Internal Medicine, New York Institute of Technology College of Osteopathic Medicine, New York City, USA; 5 Pathology, Columbia University Medical Center, New York City, USA

**Keywords:** covid-19 vaccination, acute kidney injury, autoimmune, monoclonal gammopathies, monoclonal gammopathy of renal significance (mgrs), proliferative glomerulonephritis with monoclonal immunoglobulin (mig) deposits (pgmid)

## Abstract

Proliferative glomerulonephritis with monoclonal immunoglobulin (mIg) deposits (PGNMID) is a rare glomerular disease characterized by glomerular deposits of mIg. The pathogenesis of PGNMID without circulating mIg is poorly understood but a role for aberrant immune response to infection or another exogenous stimulus has been proposed. We describe a unique case of PGNMID that presented with multiple episodes of acute kidney injury, nephritic syndrome, and hypocomplementemia, associated with self-limited febrile illnesses or COVID-19 vaccination. Monoclonal IgG lambda was detected in the serum and urine, consistent with monoclonal gammopathy of renal significance (MGRS). Consecutive kidney biopsies demonstrated evolving morphologic and immunohistologic features, with monotypic IgG lambda deposits identified only in the third biopsy. Despite the need for dialysis, renal dysfunction and hypocomplementemia resolved after each episode with corticosteroid therapy. This case illustrates infections or COVID vaccination maybe “second hits” that promote mIg deposition in PGNMID, possibly due to cytokine release by innate immune cells that promote endothelial cell injury.

## Introduction

Monoclonal gammopathies occur in around 3% of the general population over the age of 50 but undergo malignant transformation, hence the term monoclonal gammopathy of undetermined significance (MGUS) [1]. Monoclonal immunoglobulins (Ig) and/or light chains that cause kidney injury in the absence of malignancy are termed monoclonal gammopathy of renal significance (MGRS) [2]. Diagnosis of MGRS typically requires the demonstration of monotypic Ig and/or light chain deposits in the kidney by immunofluorescence microscopy (IF) [3]. 

Proliferative glomerulonephritis with monoclonal immunoglobulin (mIg) deposits (PGNMID) is a rare form of MGRS defined by glomerular deposits of mIg (usually IgG3 kappa type), glomerular hypercellularity, and electron-dense deposits that lack an organized substructure [4]. Interestingly, most cases (70%) of PGNMID lack serologic or bone marrow evidence of a B-cell clone [4]. PGNMID typically presents with mixed nephritic/nephrotic features and has a variable response to immunosuppressive and clone-directed therapy [4,5]. Morphologic and immunohistologic transformations may occur over time, making diagnosis challenging [6-8]. Coexistent infection and hypocomplementemia are not uncommon in pediatric PGNMID, suggesting a potential triggering role [9]. In addition, three cases of adult PGNMID associated with acute parvovirus B19 infection and one case associated with coronavirus disease 2019 (COVID-19) have been described [10-12].

Recently, several reports have described autoimmune phenomena and glomerulonephritis after (COVID-19 vaccination) [13-15]. We describe a unique case of PGNMID with circulating monoclonal IgG lambda protein in which acute kidney injury and the nephritic syndrome were triggered by COVID-19 vaccine and self-limited febrile illnesses. The pathophysiology of PGNMID is reviewed with a focus on the role of precipitating exogenous factors.

## Case presentation

A woman in her 50s presented with diffuse abdominal pain, generalized myalgia, nasal congestion, an erythematous maculopapular rash of the upper extremities, fatigue, fever (100.8 F), shortness of breath, and diarrhea for three days. Past history included longstanding hypertension, diverticulitis, and hypothyroidism. She had a remote history (15 years previously) of breast cancer, treated with bilateral mastectomy, radiotherapy, and chemotherapy, and no evidence of recurrence. Medications included statin and levothyroxine. She tested negative for SARS-CoV-2 RNA with a nasopharyngeal specimen. PCR tests for influenza-A and Influenza-B were negative. Hepatitis panels for Hep-A IgM, Hep Bs Ag, Hep Bs Ab, and Hep-B IgM, Hep-C Ab were negative. Her serology tests including ANA, anti-SCl-70 antibodies, anti-SSA-Ab, anti-SSB-Ab, anti-histone antibodies, and rheumatoid factor were negative. Her blood cultures and urine cultures revealed no growth. Lab values are listed in Table 1. Following a kidney biopsy, the patient underwent hemodialysis for the rise in creatinine and decreased urine output and received corticosteroid therapy (pulse dose steroids), with the prompt recovery of kidney function. 

**Table 1:** Lab findings during the hospital admissions ANA: Antinuclear antibodies

Time	First admission	12 months later	20 months later
Blood urea nitrogen (5-25 mg/dL)	70	97	60
Creatinine (0.1-1.5 mg/dL)	8.4	7.42	3.89
C3 (87-200 mg/dl)	Decreased	Decreased	Decreased
C4 (19-52 mg/dl)	Decreased	Decreased	Decreased
ANA (<1:80)	Negative	Not done	Not done
Hepatitis B (negative)	Negative	Not done	Not done
Hepatitis C (negative)	Negative	Not done	Not done
Hemoglobin / hematocrit	9.8/38	12.5/37.6	12.8/39.8
White cell count (K/μL)	13.4	7.9	6.9
Albumin (g/dL)	3.5	2	2.5
Platelets (per microliter)	-	124	97
Urinalysis	3+ protein, 11-20 RBCs, 11-20 WBCs	3+ protein, >5 RBCs, >5WBCs	2+ protein, >5 RBCs, >5WBCs
Urine protein/creatinine ratio	Not done	10,000 mg/g	500 mg/g
Immunofixation, serum	-	IgG lambda band	-
Cryoglobulin (negative)	Negative	Negative	Negative
Rheumatoid factor (negative)	Negative	Negative	Negative

The patient was well until 12 months later, when she developed fever (104° F), chills, rigors, and headache, two days after receiving a dose of COVID-19 vaccine (mRNA-1273). Chest x-ray findings were suspicious for pneumonia. Testing for SARS-CoV-2 infection was negative. A second kidney biopsy was performed. The patient again underwent hemodialysis (three sessions) for acute renal failure and received corticosteroid therapy (pulse dose steroids and subsequent oral steroids), with the recovery of kidney function.

Eight months later, she again presented with fever, myalgias, malaise, flu-like symptoms, and inability to urinate, despite adequate fluid intake. The temperature was 98.5° F, pulse 104 beats per minute, respiratory rate 18 breaths per minute, and blood pressure 113/70 mm Hg. There was bilateral 1+ pitting edema in the lower extremities and erythematous rashes on both forearms. Chest x-ray did not show any evidence of cardiopulmonary disease. Work-up for the infectious disease was negative.

Kidney biopsy findings

Microscopic and Immunohistochemical Findings

**Figure 1:**
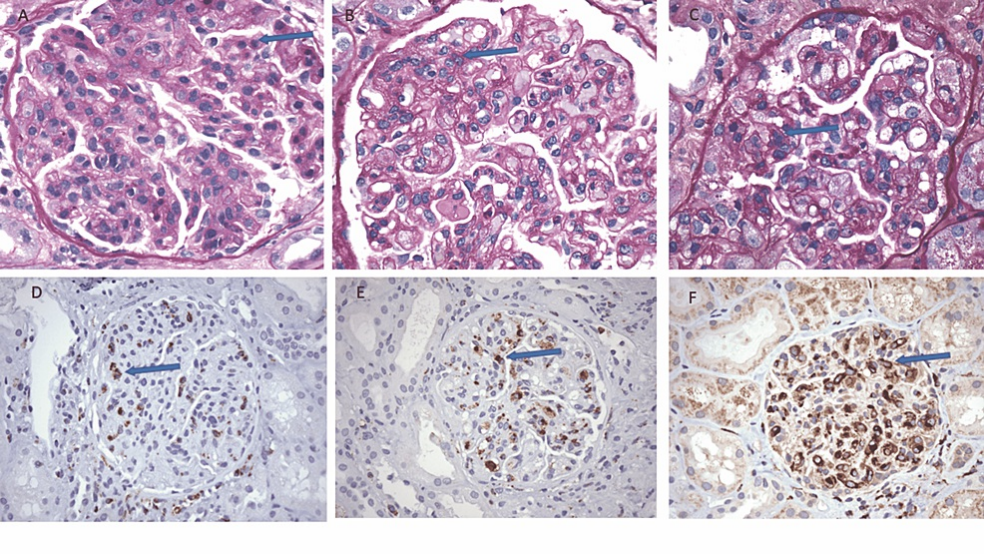
Pictures in the top row demonstrating endocapillary hypercellularity with endothelial cell swelling with Periodic acid-Schiff (PAS) stain (arrows). Pictures in the bottom row demonstrating macrophage infiltrates with CD68 Immunohistochemical stain (arrows). (A, D) First kidney biopsy. (B, E) Second kidney biopsy. (C, E): Third kidney biopsy

The first kidney biopsy showed glomeruli with diffuse endocapillary hypercellularity, endothelial cell swelling and (Figure 1A) and CD68+ macrophage infiltrates (Figure 1D). The second kidney biopsy showed diffuse endocapillary hypercellularity/endothelial swelling with segmental membranoproliferative features (Figure 1B), macrophage infiltrates (Figure 1E), and segmental subendothelial hyaline deposits, and patchy acute tubular injury. The third kidney biopsy revealed diffuse endocapillary hypercellularity, segmental hyaline deposits, and segmental duplication of glomerular basement membranes (Figure 1C). Numerous CD68+ macrophages were seen (Figure 1F). There was a patchy acute tubular injury but the degree of tubulointerstitial scarring and inflammation remained minimal.

Immunofluorescence Staining

**Figure 2:**
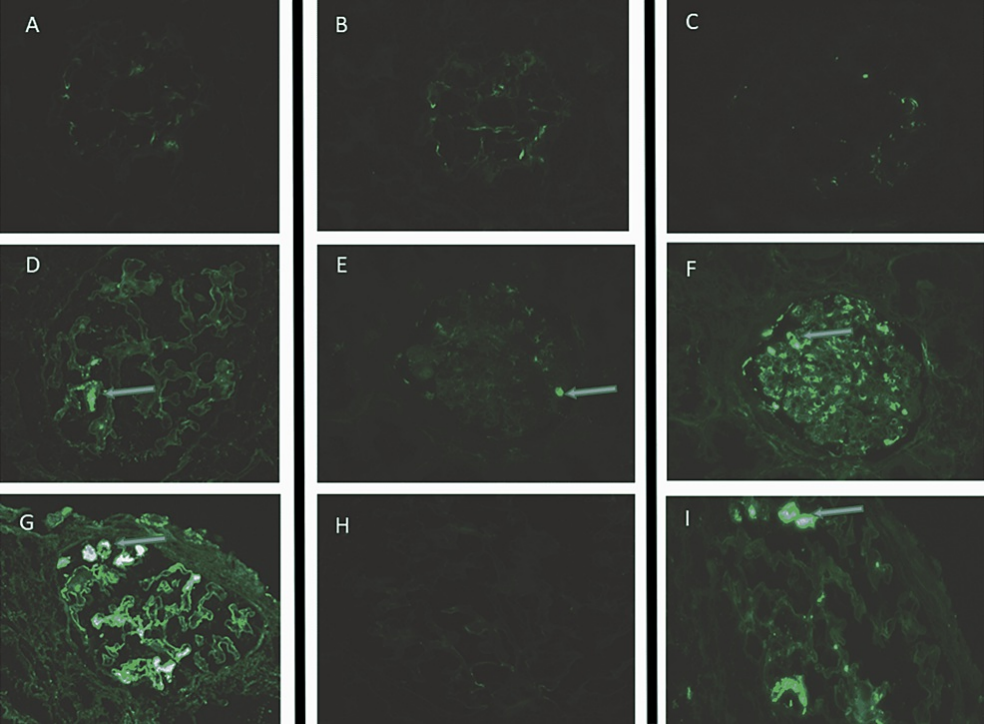
Immunofluorescence (IF) demonstrating deposited immunoglobulins and light chains (arrows). (A-D) First kidney biopsy demonstrating trace to 1+ segmental tuft staining for IgM immunoglobulins, kappa, lambda light chains, and C3. (E, F) Second kidney biopsy demonstrating 1+ mesangial and capillary wall staining for IgM immunoglobulins. (G-I) Third kidney biopsy showing IgG lambda.

The first kidney biopsy results revealed the patchy acute tubular injury and minimal interstitial fibrosis, edema, and inflammation. Immunofluorescence (IF) demonstrated trace to 1+ segmental tuft staining for IgM, kappa, lambda, and C3 (Figures 2A-2D). The second kidney biopsy results showed 1+ mesangial and capillary wall staining for IgM only (Figures 2E, 2F). The third kidney biopsy showed segmental capillary wall staining for IgG and lambda (Figures 2G-2I). IF staining for IgG heavy chains showed no clearcut staining for IgG1, IgG2, IgG3, or IgG4, and was non-contributory. However, staining for heavy and light chains revealed positivity for IgG lambda but not IgG kappa, IgM kappa, or IgM lambda. 

Electron Microscopy Findings

**Figure 3:**
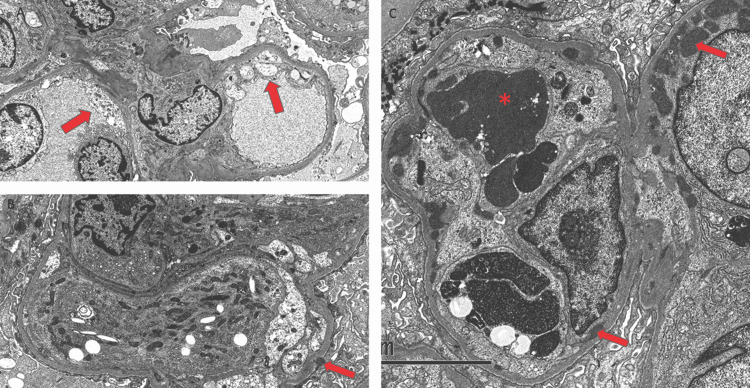
Electron microscopy demonstrating subendothelial electron-dense deposits (arrows). (A) First kidney biopsy showing endothelial cell swelling. (B) Second kidney biopsy showing a few mesangial and capillary wall immune-type electron-dense deposits. (C) Third kidney biopsy showing multiple large subendothelial electron-dense deposits, some of which appeared to be undergoing ingestion by intracapillary leukocytes.

The first kidney biopsy revealed endothelial cell swelling, but no electron-dense deposits were identified (Figure 3A). A diagnosis of endocapillary proliferative glomerulonephritis of unclear etiology was made. The second kidney biopsy showed a few mesangial and capillary wall immune-type electron-dense deposits, without an organized substructure (Figure 3B). The third kidney biopsy showed multiple large subendothelial electron-dense deposits, some of which appeared to be undergoing ingestion by intracapillary leukocytes (Figure 3C).

Bone marrow biopsy

Following the second kidney biopsy, serum and urine protein electrophoresis and immunofixation revealed the presence of monoclonal IgG and lambda light chain. A bone marrow biopsy showed 8% plasma cells with IgG lambda staining.

The first two biopsies showed proliferative glomerulonephritis with prominent endothelial swelling (“endotheliopathy”), numerous histiocytes, and no immune deposits. The major diagnostic considerations were acute thrombotic microangiopathy, cryoglobulinemic glomerulonephritis, and histiocytic glomerulopathy. However, lab investigations did not support any of these diagnoses. Subsequently, the discovery of a monoclonal IgG lambda in the serum and urine, and bone marrow biopsy showing 8% plasmacytosis, led to the consideration of MGRS. However, only the third kidney biopsy showed clearcut monotypic IgG lambda deposits, consistent with PGNMID. Repeat IF after pronase digestion performed on the first two kidney biopsies revealed intraluminal staining for IgG and lambda, consistent with circulating mIg, but no clearcut mesangial or capillary wall staining, providing evidence against masked mIg deposits. Thus, the immunohistologic features of this case evolved from no detectable deposits initially, to scant electron-dense deposits in the second biopsy, to definitive IgG lambda deposits in the third biopsy. In summary, the most parsimonious interpretation is that glomerular injury in all three biopsies was caused by the presence of monoclonal IgG (i.e., MGRS), even though this diagnosis could only be established with certainty in the third biopsy.

Treatment and follow-up

Following each biopsy, the patient received intravenous steroids and dialysis, and kidney function improved rapidly, with serum creatinine returning to a new baseline of 1.3 to 1.6 mg/dL. At the last follow-up (four months after the third biopsy), serum creatinine was 1.6 mg/dL and the patient was asymptomatic.

## Discussion

MGRS comprises a broad variety of renal diseases that result from intrinsic nephrotoxic properties of various monoclonal immunoglobulins and their subunits. In this case of PGNMID, the clinical manifestations of AKI and transient hypocomplementemia were associated with self-limited febrile illnesses and COVID-19 vaccination. Although ongoing IgG lambda deposition between episodes of AKI could not be excluded, the prompt recovery of kidney function with steroid therapy each time and lack of progressive scarring suggests intermittent IgG deposition. This unusual clinical course supports a pathogenic role for exogenous triggers (“second hits”) in PGNMID, including the COVID-19 mRNA vaccine. The findings of endothelial injury and infiltrating histiocytes in all three biopsies may reflect cytokine release from innate immune cells, and multiple bouts of capillary wall injury may have set the stage for the eventual precipitation of mIg, permitting classification as PGNMID.

Of note, the prominent macrophage infiltrates might account for the paucity of immune deposits in the first two biopsies, as sometimes occurs in cryoglobulinemic glomerulonephritis. However, ultrastructural features of cryoglobulin were not identified and there were no other clinical signs of cryoglobulinemic vasculitis in this case.

Why some cases of PGNMID show false negative staining by routine immunofluorescence microscopy on frozen tissue is unclear [16]. In the case of light chain proximal tubulopathy, the intracellular location of the monoclonal light chains may limit binding by anti-light chain antibodies. In some cases of PGNMID, the tertiary or quaternary structural characteristics of the mIg may impede the binding site of the anti-IgG antibody, which is then “unmasked” by pronase digestion. The higher antibody concentration used for pronase versus routine IF might also explain why relatively sparse or cryptic monoclonal Ig deposits are detectable only after pronase digestion [7]. In this case, we can only speculate that the presence of exuberant macrophage-rich glomerular infiltrates and vigorous phagocytosis may have contributed to the sparsity of Ig deposits seen in the earlier biopsies, resulting in false-negative immunofluorescence staining for IgG.

## Conclusions

This report illustrates that infections and COVID-19 vaccination may be triggers for the clinical expression of MGRS. Management should be patient-tailored, and immunosuppressive therapy may be beneficial. In general, COVID-19 vaccines are safe and effective and have played a major role in curtailing the novel coronavirus pandemic. However, adverse side effects, including exacerbation of underlying kidney disease, occur occasionally but should not lead to vaccine hesitation, since the benefits of vaccination greatly outweigh the risks of COVID-19 infection in patients with preexisting kidney disease.
